# Inferred vegans in studies not designed to assess vegan status may be largely artifacts

**DOI:** 10.1038/s41538-026-00985-3

**Published:** 2026-07-21

**Authors:** Christian Koeder, Maximilian Andreas Storz

**Affiliations:** 1 Independent Researcher, Ellwangen, Germany; 2 Department of Internal Medicine II, Centre for Complementary Medicine, Medical Center – University of Freiburg, Faculty of Medicine, University of Freiburg, Freiburg, Germany

**Keywords:** Diseases, Health care

## Abstract

Recent studies from China and India, none designed to assess vegan status, reported unusually large vegan samples (*n* = ~1000–8000) and prevalences (~1–9%). These were mostly inferred from short food frequency modules or short-term dietary assessment (e.g., 1 month). Many inferred vegans were socioeconomically marginalized or socially excluded. This perspective argues that the reported prevalences largely result from underassessment of intake and misclassification rather than adherence to vegan diets.

## Scope

The present perspective reviews three large cohort studies from China – the China Kadoorie Biobank (CKB)^1^, Chinese Longitudinal Healthy Longevity Survey (CLHLS)^2,3^, and Tianjin Chronic Low-grade Systemic Inflammation and Health (TCLSIH)^4^ studies – and three rounds of the National Family Health Survey (NFHS) in India^5–8^ and argues that overall dietary assessment in these studies was too shallow (in most studies) or too short-term (TCLSIH, NFHS-4 children) to allow the assessment of participants’ vegan diet status.

## Introduction

### Definition of vegan diets

Vegan diets are defined by the exclusion of all animal products^9–12^. The founders of the Vegan Society (UK) coined the terms vegan and veganism in the mid-1940s^13^. This organization is therefore widely regarded as an authority on defining these terms. The Vegan Society’s definition of veganism states: “Veganism is a philosophy and way of living which seeks to exclude—as far as is possible and practicable—all forms of exploitation of, and cruelty to, animals for food, clothing or any other purpose; and by extension, promotes the development and use of animal-free alternatives for the benefit of animals, humans and the environment. In dietary terms it denotes the practice of dispensing with all products derived wholly or partly from animals”^14^. This definition provides clarity that vegan diets exclude all animal-source ingredients. This definition also suggests that vegan diets are not one specific diet, which allows the conclusion that vegan diets can be centred around healthful or unhealthful foods and that they may lack certain nutrients (e.g., vitamin B12) or provide these (e.g., through vitamin B12 fortification). Moreover, this definition describes vegan diets as a consequence of the philosophical view termed veganism, i.e., a stance against animal exploitation. Seen in this way, vegan diets can be defined as conscious and voluntary abstention of animal product consumption. In this way, involuntary abstention from all animal products would not be defined as vegan dietary practice. For example, Ramadan fasting would not be considered a vegan diet from dawn to sunset, and dying of starvation would not be seen as a vegan diet, although these scenarios exclude all animal product ingestion. A further, theoretical example includes are individuals who, while not dying of starvation, are so monetarily poor that they can neither afford any type of animal product nor any type of food containing animal products, resulting in an involuntary vegan diet. It may be argued that such individuals should not be terms vegans, as involuntary food deprivation is not related to the philosophical view termed veganism (as described in the Vegan Society’s definition). Conversely, it may be argued that such individuals are indeed vegans as their diet is devoid of all animal products. To avoid this problem, some experts recommend explicitly defining vegan diets as voluntary abstention from all animal products^15^. Nevertheless, this is a semantic distinction. The more pertinent question in nutritional sciences is (1) whether population segments involuntarily adhering to diets devoid of all animal products truly exist and (2) whether, if they do exist, such diets are globally deficient diets that are incomparable to voluntarily chosen vegan diets in any part of the world (Table 1).

**Table 1:** Voluntary vegan diets vs. poverty-related dietary restriction of ASF

	Voluntarily chosen ASF-free diet	Severe restriction of ASF due to poverty	
**Semantic distinction**	Contains no ASF at all	Contains no ASF at all	Theoretical concept, unlikely to be observed in practice
	“True vegan diet”, voluntarily vegan	Involuntarily vegan	
	Typically considered a vegan diet by official definitions, vegan organizations, and the vegan community	Not typically considered a vegan diet by official definitions, vegan organizations, or the vegan community	
**Dietary distinction**	Contains no ASF at all	Diet low in ASF (severe poverty diet, not vegan)	
	--	Starvation (no food intake at all: not a dietary pattern) *	

*Similarly, water-only fasting or severely restrictive diets (e.g., bread and water only) are not typically considered a vegan dietary pattern.

*ASF* animal-source foods and foods containing animal-source ingredients.

### Difficulties in ascertaining the prevalence of vegan diets

Two broad categories for the assessment of the prevalence of vegan diets can be described: (1) Prevalence is assessed based on multiple-choice questions about food group intake: typically using questions such as “Do you consume meat/fish/eggs/dairy?”, with answer options such as “daily/weekly/less than weekly/never” (hereafter: intake-based) ^16^. (2) Prevalence is assessed based on self-reported dietary category (hereafter: self-identification): typically using questions such as: “Do you adhere to an omnivorous/a lacto-ovo-vegetarian/a vegan diet?”, with the answer options “yes/no”^17^. The latter method (self-identification) typically results in a higher prevalence, as some individuals report a vegan diet, although they consume certain animal products at least occasionally. Notably, neither of the two methodological approaches can determine strict vegan diets, as many food preparations (e.g., bread, cake, soup) are based on non-animal-source ingredients but are non-vegan. Consequently, a combination of both approaches may be preferrable, depending on the research question.

### Reported prevalence of vegan diets

The prevalence of vegan diets among the general adult population is likely highest in relatively financially affluent regions, including the Nordic countries, the UK, parts of central Europe, and Israel. Although many scientific publications do not distinguish between intake-based prevalence and prevalence based on self-identification^18–23^, this distinction appears crucial as they capture different groups, with estimates based on self-identification often notably higher. Additionally, the prevalence of vegan diets in many regions of the world appears to have continuosly increased from the 2000s to the 2010s and 2020s^18^. Therefore, when comparing reported prevalences between studies the year of assessment should be considered. Estimated intake-based vegan diet prevalence in the UK, Switzerland (both 2000s–2010s), and Spain (2020) was 0.1%^16,24,25^, while in New Zealand (2018–2020) it was 0.7%^26^. Estimated vegan diet prevalence based on self-identification in parts of Western Europe (2020 s) was about 2–4%^21,27–31^. These data from general adult populations can be contrasted with the two large Western cohort studies that include several thousands of vegans: EPIC-Oxford (UK) oversampled vegans and has a vegan diet prevalence of about 3.7%^1^. The Adventist Health Study 2 (AHS-2; USA and Canada) has a vegan diet prevalence of about 8.1%^1^. Its participants are Seventh Day Adventists with a known high prevalence of vegan diets. Additionally, an intake-based estimate from 25 European countries (2020 s) suggests a prevalence of plant-forward (i.e., low-meat omnivorous) diets of 1.6%^32^, which indicates that at the general European adult level, the prevalence of vegan diets is likely considerably lower than 1.6%. For most countries, data on vegan diet prevalence are largely lacking^33,34^, but it appears likely that the intake-based prevalence of vegan diets in most low- and middle-income countries falls notably below 1%^35^.

### Implausibly high vegan prevalences reported in recent studies from China and India

The present perspective argues that the existence of vegans in population-based cohort studies or surveys cannot be extrapolated from the intake of animal-source foods (ASF) if dietary intake assessment was superficial (e.g., with food frequency modules with 8–13-items in total^1–3,5,6,8^) (Table 2) and/or too short-term (e.g., 24  h^7^ or 1 month^4^) (Table 3). This applies even more strongly to studies not intended and designed to assess vegan status (Table 4, Table 5). For example, ASF intake assessment is insufficient, if intake of only certain dairy products (e.g., milk, yoghurt) is assessed^1,36^ (Table 3) or if intake frequency categories are relatively imprecise, e.g., occasionally, compared to never, weekly, daily^37–39^ (Table 5). In the latter case, some participants may understand “never” as less than weekly (Table 5)^40^. Similarly, diet being assessed only for the previous month ^4^ (Table 4) is likely a contributing factor to the high vegan diet prevalence in TCLSIH.

**Table 2:** Population-based studies reporting vegans in China and India

Study	Vegan definition	Study year	Group	Age (years)	Vegan prevalence (%)	Vegans (n [N])	Food items in FFQ (n)	Health effects
CKB ^1^	“Never/ rarely”: meat, poultry, fish, seafood, fresh eggs, dairy products (milk, yoghurt)^36^	2004/ 2008	Adults	30–79	1.0	5,110 [510,145]	12 items^36,113^	None rep.
		2013/ 2014	Adults	Not rep.; ~35–89	1.2*	302 [Not rep.; ~25,167]	12 items (incl. milk powder, cheese, and milk as a drink)^114^	
		Vegan at both 2004/ 2008 and 2013	Adults	--	0.1*	~613 [Not rep.; ~613,000]		
CLHLS (-2018)^2^	“Rarely or never”: meat, fish, eggs, milk products	1998–2018	Adults	65–122	3.9	1084 [27,917]	13 items	Harm
CLHLS (-2014)^3^	Dairy intake not assessed; “no [..] eggs, fish, seafood, or meat”	1998–2014	Adults	60–79	2.9	83 [2888]	12 items	Harm
TCLSIH^4^	“Nearly never” in “previous month”: dairy, meat, meat products, blood, organs, fish, seafood, eggs, preserved eggs	2013–2019	Adults	40–50	8.6	2288 [26,490]	100 items †	Neutral (benefit in women)
NFHS-5^5^	Not rep.; according to gov. report: “never”: milk, curd, eggs, fish, meat^37^	2019–2021	Non-preg. women	15–49	1.0	8332 [817,381]	10 items	Harm
NFHS-4^6^ ‡	“Never”: milk, curd, eggs, fish, meat^38^	2015–2016	Mothers of pre-school.	Not rep.	1.1	~2454 [223,040]	10 items	Children: harm (x4); neutral (x2) §
NFHS-3^8^	“Never” milk, curd, eggs, fish, meat ¶	2005–2006	Adults	20–49	1.6 ‖	2560 [156,317]	8 items	Neutral
NFHS-4 child^7^.	Exclusively: cereals, tubers, legumes, nuts, fruit, vegetables; no flesh foods, eggs, dairy, infant formula, foods made with oil/fat^38^	2015–2016	Young children	0.5–1.9 (6–23 months)	7.8	452 [5,772]	21 items (not FFQ) #	Neutral (x2); harm (x1)**

*In 2013, 65% and 12% of baseline (2004) vegans were reclassified as omnivores and vegans, respectively, coinciding with improved socioeconomic status and additional assessment of cheese and milk powder in 2013^1^, suggesting underassessment at baseline.

†It was not reported which food items assessed were considered vegan.

‡The same authors reported that in the National Sample Survey (2011–2012) in India (a survey to measure per capita availability of food groups at the household level) showed that 5.9% of households were “vegan household[s]” (*n* = 5958); this classification was based on a 30-day diet recall module^6^.

§ harm: stunting (not at 24–59 months, contrary to the authors’ prior hypothesis); wasting (unclear when stratified by age, contrary to the authors’ prior hypothesis), anaemia (not when stratified by age); diarrhoea (not at 0–5 months, when most children were breastfed); neutral: height-for-age and weight-for-height. The authors state that their data preclude causal inferences^6^.

¶ The publication states that the vegans likely consumed butter and ghee and that the concept of vegan diets may not be correctly communicated in India^8^.

‖ This prevalence was cited by others as nationally representative^115^.

# Maternal 24-hour recall

** Neutral/non-significant (stunting, wasting); harm (underweight)^27^

*Child.* children, *CKB* China Kadoorie Biobank, *CLHLS* Chinese Longitudinal Healthy Longevity Survey, *FFQ* food frequency questionnaire or short food intake module, *gov.* government, *N* all dietary groups combined, *NFHS* National Family Health Survey, *preg.* pregnant, *rep.* reported, *school*. schoolers, *TCLSIH* Tianjin Chronic Low-grade Systemic Inflammation and Health

**Table 3:** Potential underassessment of ASF intake in the cited cohort studies and surveys

Country	Study	ASF categories assessed	ASF likely missed	Implications for classification
**China**	CKB ^1^ *	ASF consumed less than once per month †: ●meat ●poultry ●fish ●seafood ●fresh eggs dairy products (milk, yoghurt)^36^	●flesh foods other than meat/poultry or fish/seafood: organ meats/offal (see TCLSIH), amphibian/reptile meat ●blood (see TCLSIH), blood tofu ●insects ●eggs other than “fresh eggs”, e.g., processed eggs (preserved, salted, powdered, or liquid eggs); egg-containing foods, e.g., cakes, pastries, bread, cookies, egg noodles, mayonnaise, sauces, batter ●**dairy products other than milk or yoghurt** ●**“Soybean products” containing ASF**: tofu cooked in meat broth/lard or with pieces of meat/ground meat added; stinky/fermented tofu with fish/shrimp paste/lard; tofu snacks with meat essence/extract, fish sauce, or lard; soya milk with whey powder/milk solids ●**preserved vegetables containing ASF**: preserved vegetables with shrimp brine/paste, fermented small shrimp, fish sauce, dried fish powder, lard, small pieces of meat, ground pork, meat stock, chicken essence ●drinks containing ASF: milk teas, chocolate/coffee drinks with dairy; bone broth drink or other medicinal tonics; fish oil ●non-vegan ingredients in processed foods^36^	Superficial dietary assessment, therefore unable to assess vegan diets
	CLHLS (-2018)^2^	ASF consumed less than once per month †: ●meat ●fish ●eggs ●milk products	●**dairy products (before 2008)** ●ASF items not assessed (see CKB above)	Superficial dietary assessment, therefore unable to assess vegan diets
	CLHLS (-2014)^3^	●meat ●fish ●seafood ●eggs	●**dairy products** ●ASF items not assessed (see CKB above)	Very superficial dietary assessment, therefore unable to assess vegan diets
	TCLSIH ^4^	●meat ●meat products ●blood ●organs ●fish ●seafood ●eggs ●preserved eggs ●dairy	●**ASF outside of the “previous month” window** ●ASF items not assessed (see CKB above)	Short-term dietary assessment, therefore unable to assess long-term vegan diets
**India**	NFHS-5^5^ ‡	●chicken or meat ●fish or chicken or meat ●eggs ●milk or curd^37,38^	●**dairy products other than “milk or curd”**: paneer, ghee, butter, Western-style cheese/cheese powder, khoya/mawa (reduced milk solids), condensed milk (used in burfi, rabri, and other sweets), lassi/buttermilk, paneer by-products/whey, tea/coffee with milk ‖ ●**fried foods** #: fried chicken, fish fry, burgers, and mutton/lamb pakora; fried foods containing ghee, dairy, egg, or meat-based flavourings, including samosas, pakoras, vadas fried in ghee and/or filled with paneer, meat, or eggs; instant noodles or fried snacks containing milk solids, whey, paneer, cheese powder, or hydrolysed animal protein ●flesh foods potentially not perceived as meat, chicken, or fish: prawns, shrimps, crabs, duck meat, organ meats, dried fish, insects, and foraged ASF ●eggs potentially not perceived as eggs: duck, quail, Guinea fowl, or pigeon eggs	Superficial dietary assessment, therefore unable to assess vegan diets
	NFHS-4^6^ §¶			
	NFHS-3^8^	●chicken or meat ●fish or chicken/meat ●eggs ●milk or curd^39^		
	NFHS-4 children, 6–23 months old^7^ ¶**	●meat ●poultry ●organ meats ●fish ●shellfish ●eggs ●milk ●cheese ●yoghurt ●other milk products ●infant formula ●foods made with oil/fat^38^	●**ASF eaten outside of the 24-hour window**: “in the day or night preceding the interview”^38^ ●ASF items not assessed (see above) ●non-vegan ingredients in bread, noodles, soup, broth, porridge, gruel, or fortified baby food)^7,38^	Superficial dietary assessment, therefore unable to assess vegan diets. At the same time overly restrictive definition of vegan diet: exclusively cereals, tubers, legumes, nuts, fruit, vegetables^7^. The vegan children reportedly did not receive foods made with oil/fat or infant formula^38^. Exclusion of ASF, infant formula, and foods with oil/fat appears to suggest poverty and a severely deficient infant diet, not a voluntarily chosen vegan diet.

Bold text in the right column highlights aspects deemed particularly relevant.

*The food frequency module was validated ^1^ but not validated to assess vegan status.

†as in AHS-2^1,101^.

‡When FFQ-based intake data in the CKB study were compared with 24-hour recalls, the FFQ overestimated the prevalence of “never/rarely” consuming red meat by 100–200%^116^.

§The NFHS-5 government report suggests that data on mortality estimates “depends on the mother’s ability to recall all of the children she has given birth to, as well as their birth dates and ages at death”^37^. This appears to support the assumption that some participants (particularly if vitamin B12 deficient) may not have accurately recalled what they “never” ate.

¶ “milk or curd” interpreted by the authors as equivalent to dairy products^6^; authors^6^ and journal editors (previous submission; personal communication: October 2025) stated that vegan mothers were correctly classified as vegans.

‖ The authors state that the term “vegan” may not be correctly asked/interpreted/self-reported in India and that the vegans in NFHS-3 probably consumed butter/ghee/ honey^8^.

#Fried foods were included in the NFHS-4 and NFHS-5 surveys (not NFHS-3); the cited studies appear to have assumed that fried foods are vegan^5–7^.

**Importantly, the authors correctly highlight that a minimum diversity diet, per World Health Organization guidelines, is inadequate if it only includes unfortified plant food groups. The authors also note that analyses include only surviving children, which may underestimate the risks of severely deficient diets.

*AHS-2* Adventist Health Study 2, *CKB* China Kadoorie Biobank, *CLHLS* Chinese Longitudinal Healthy Longevity Survey, *NFHS* National Family Health Survey, *TCLSIH* Tianjin Chronic Low-grade Systemic Inflammation and Health.

**Table 4:** Vegan status assessment in AHS-2 (USA and Canada) versus cited studies from China

AHS-2 and cohorts in China: vegan diet defined as ASF intake less than once per month					
	AHS-2	CKB	CLHLS (1998–2018) *	CLHLS (1998–2014) *	TCLSIH
**Recruitment**					
Population with known high vegan prevalence †	Yes	No	No	No	No
**Study aim**					
Assessment of health outcomes in vegans	Yes	No	No	No	No
**Vegan status assessment**					
Self-identification	No	No	No	No	No
Detailed FFQ	Yes	**No**	**No**	**No**	Yes
Long-term diet	Yes	Yes (past year)	**No**^2^	**No**^2^	**No**
ASF intake criterion for vegan classification	<monthly	“Never/rarely” (other categories: daily, 4–6 days/week, 1–3 days/week, monthly)^36^	**Dairy intake not assessed before 2008**; “Rarely or never” (less than: sometimes or occasionally, always or almost every day [before 2008]; occasionally, at least once per month [from 2008]); “rarely or never” was in response to the question: how often [do you] eat […] **at present**”?	**Dairy intake not assessed**; “never”	“Nearly never” in “**previous month**”
Asked about meat and fish intake in general	Yes	Yes	Yes	Yes	Yes
Asked about egg intake in general	Yes	**No (limited to fresh eggs)**^36^	Yes	Yes	Yes
Asked about dairy intake in general	Yes	**No (limited to milk and yoghurt)**^36^	**No (before 2008)**; yes (from 2008)	**No**	Yes
Mention of “vegan” to participants	No	No	No	No	No

Bold text highlights aspects deemed particularly relevant.

* Both studies are from the same research group^2,3^.

† Adherence to a vegan diet in AHS-2 appears stable over time^101^.

*AHS-2* Adventist Health Study 2, *CKB* China Kadoorie Biobank, *CLHLS* Chinese Longitudinal Healthy Longevity Survey, *TCLSIH* Tianjin Chronic Low-grade Systemic Inflammation and Health.

**Table 5:** Vegan status assessment in EPIC-Oxford (UK) versus cited studies from India

EPIC-Oxford and NFHS (India): vegan diet defined as “never” consuming ASF					
	EPIC-Oxford	NFHS-5	NFHS-4 *	NFHS-3	NFHS-4 children
**Recruitment**					
Via vegetarian and vegan organizations	Yes	No	No	No	No
Designed to recruit as many vegans as possible	Yes	No	No	No	No
**Study aim**					
Assessment of health outcomes in vegans	Yes	No	No	No	No
**Vegan status assessment**					
Self-identification	No	No	No	No	No
Detailed FFQ in case of ambiguity	Yes	**No**	**No**	**No**	**No**
Long-term diet	Yes	Yes	Yes	Yes	**No**
ASF intake criterion for vegan classification	“Do you eat any [meat, fish, eggs, dairy; with examples]?” †	“Never” (less than: occasionally, weekly, daily)^37^; thus, **“never” may mean <weekly**. ‡			**Not “in the day or night preceding the interview”** ¶
Asked about meat, fish, and egg intake in general	Yes	Not reported; according to the government report: yes	Yes	Yes	Yes
		**Fried foods were considered vegan**			
Asked about dairy intake in general	Yes	**No (limited to milk and curd)**			Yes
Mention of “vegan” to participants	No	No	No	No	No

Bold text highlights aspects deemed particularly relevant.

*The publication not only claims that participants classified as vegans were actual dietary vegans but also that NFHS-4 data allowed for determination of “the prevalence of veganism among Indian mothers” in different states in India^6^ [Supplemental Figure 3 in reference]; however, veganism (as a sociocultural practice) would more explicitly exclude involuntary vegans.

†Vegans were classified based on four questions on consumption of meat, fish, dairy, or eggs (including eggs in baked goods), with examples provided for each food group^117^. Ambiguous cases were confirmed using FFQ data covering 130 food items plus additional questions^118^ (personal communication, P. Appleby, October 2025).

‡The NFHS-5 government report states: “Very few women consume chicken, meat, fish, or eggs daily, although about one-third of women consume these types of food weekly”^37^. thus, consuming any ASF less than weekly may be common, but alternating consumption of different ASF on different days of the week may be more common.

¶ NFHS-4: 6–30% of children aged 6–23 months were reported to not have consumed foods from the “Meat, fish, poultry, and eggs” category “in the day or night preceding the interview” (2015–2016)^38^ [Table 10.9 in reference]. This percentage was 8–30% in NFHS-5^37^. To classify these children as vegan^7^ appears unjustified.

*EPIC* European Prospective Investigation into Cancer and Nutrition, *NFHS* National Family Health Survey.

Furthermore, as most individuals do not appear to conceptualize their food in terms of isolated food groups (as assessed in simple food modules), underassessment of ASF intake in such studies appears probable^41^. This precludes any meaningful assessment of vegan status.

Furthermore, in epidemiological research, it is imperative to declare a study’s prespecified objectives, hypotheses, and primary parameters. Therefore, in studies in which vegan diets were not part of a prespecified study plan, any observed associations in this regard must be considered hypothesis-generating and should explicitly be described as exploratory, even if ASF intake is assessed in detail and long-term.

In addition, observed prevalences of vegan diets should be compared with existing knowledge. When in privileged regions, such as Western Europe or New Zealand, intake-based vegan diet prevalence is reported to have been <1%^16,24–26,42^, with prevalence estimates based on self-identification of about 1–2% in Germany^17,29,43,44^ and the USA^45^, then reports of unexpectedly large vegan samples (e.g., n > 5000)^1,5^ and unexpectedly high prevalences of 1–9%^1–8,46,47^ (Table 2) in China and India warrant scrutiny.

### Harm from vegan diets

This perspective does not dispute reported associations between vegan diets and health outcomes^48^, nor that such diets can be severely deficient. The present authors have consistently argued that unsupplemented, unfortified (USUF) vegan diets pose serious risks^13,48^: such diets have caused severe, potentially irreversible nerve damage^49,50^, other grave health consequences, and, in some cases, infant deaths^13,48^.

### USUF vegan diets are nutritionally implausible

The lack of vitamin B12 in USUF vegan diets – although exceptions are possible^51,52^ – is one argument why the high prevalence of vegan diets in the described studies (Table 2), including among socioeconomically highly disadvantaged population segments (Table 6), appear implausible.

**Table 6:** Socioeconomic factors in vegans that suggest potential misclassification

Country	Studies	Indicators		
		Dietary/health	Socioeconomic	Demographic
**China**	CKB^1^ *	Vegans: **●consumed much less food**: no ASF; additionally, **lowest intake of** refined grains, **total grains**, vegetables, and fruit (legume and nut intake not assessed) †	Vegans: **●**lower income (data not reported) † **●lowest university degree prevalence** (**0.3%** vs. 6.0% in OMN) †	• Vegans: **mostly from two rural regions** (Gansu and Henan) †
	CLHLS (-2018)^2^ *	Vegans: **●45% underweight** † **●**21% current smokers ‡§ **●**12% heavy drinkers ‡§	Vegans: **●49% in the lowest income category** †‡ **●31% reported insufficient financial support** †‡ **●75% with 0 years of education** †‡	Vegans: **●46% living in rural residence** †‡ **●mean age: 87 years** ‡
	CLHLS (-2014)^3^ *	See above	See above	See above
	TCLSIH^4^ *	Vegans: **●**95% did not take nutritional supplements †‡¶	Vegans: Income and education not reported.	--
**India**	NFHS-5^5^	—	Vegan prevalence highest among: **●the poorest (almost 25%)** † **●**women with primary or secondary (but no higher) education and **men with no education (less than primary)** †‡	Vegan prevalence highest among: **●**scheduled tribes (Adivasis; historically disadvantaged indigenous communities), with **>35% of vegans belonging to scheduled castes** (Dalits; historically: “untouchables”) **or scheduled tribes** †‡ **●rural households (>** **80%)** ‡ **●**15–19-year-old women and 40–49-year-old men ‡ **●**Sikh women ‡ and Hindu men **●**Women (1.1%; compared to men: 0.4%) †
	NFHS-4^6^ ‖	—	Vegan prevalence: **●**decreased from the poorest to the richest quintile, with **>** **30% of vegans in poorest quintile** †	Vegan prevalence: **●**highest among scheduled tribes, with **almost 50% of vegans belonging to scheduled tribes or scheduled castes** †# **●**higher in rural than in urban areas, with **>** **80% of vegans residing in rural households** ‡ **●**No difference in Cattle ownership between households with vegan or OMN mothers ‡
	NFHS-3^8^	—	Vegans: **●23% were in the lowest wealth quintile** † **●47% had less than primary school education** †	Vegans: **●30% belonged to scheduled castes or scheduled tribes** † **●70% resided in rural areas** ‡
	NFHS-4 children, 6–23 months old^7^		**●**Household wealth positively associated with dairy intake †	--

Bold text highlights aspects deemed particularly relevant, suggesting that many individuals classified as vegans were not vegans by choice.

* No information regarding religion reported. CLHLS included participants from across China^2^.

†These characteristics suggest poverty and/or underreporting among those categorized as vegans.

‡At the group level, this appears unlikely among vegans motivated by views related to animal ethics, environmental protection, or health.

§At the group level, this appears unlikely among vegans motivated by Buddhism in China.

¶This suggests mostly USUF vegan diets.

‖The authors suggest that lacto-vegetarian diets are mostly found among upper caste Hindus who also tend to be wealthier and more educated^6^.

#Lacto-vegetarian prevalence was lower among scheduled castes and increased with household wealth. The authors had hypothesized that vegetarians may be wealthier than non-vegetarians. Lacto-vegetarians were more likely to live in households with better hygienic circumstances. The authors note that ASF are important in diets of tribal populations.

*CKB* China Kadoorie Biobank, *CLHLS* Chinese Longitudinal Healthy Longevity Survey, *NFHS* National Family Health Survey, *OMN* omnivores, *TCLSIH* Tianjin Chronic Low-grade Systemic Inflammation and Health, *USUF* unsupplemented, unfortified.

If some populations are so monetarily poor that their diets are completely ASF-free, which may technically be described as involuntarily vegan diets, then these would almost certainly be USUF vegan diets. This is confirmed by one cited publication which (reporting on NFHS-4) states that ASF are “the only dietary source” of vitamin B12^6^.

Although individuals who *involuntarily* subsist on USUF vegan diets for several years may exist and may have been captured in the described studies (Table 2), the existence of such diets in larger numbers of individuals appears implausible due to nutritional defects. Even the cheapest ASF would be inaccessible, leaving diets severely restrictive (e.g., only white rice; insufficient calories) and globally deficient (Table 1).

Apart from the issue that research testing whether globally deficient diets are harmful can be redundant, the relatively moderate health problems reported for these USUF vegan diets (Table 2) appear implausible. For example, despite large sample sizes, NFHS-4 data showed no significant association of maternal vegan diet with stunting (ages 0–5 and 24–59 months) or wasting (ages 0–59 months)^6^, nor of child vegan diet with stunting or wasting (ages 6–23 months) ^7^. These results were also highlighted a recent ESPGHAN Nutrition Committee position paper on vegan children, which reports that NFHS-4 data showed “a significantly higher risk of stunting (according to WHO growth references) among vegan infants and toddlers […], compared to vegetarians in crude models, but the association lost significance after adjustment”^27^. While adequately supplemented vegan diets for children may be safe^53^, the NFHS-4 data may be misinterpreted to suggest that USUF vegan diets in young children are only associated with moderate harm, underestimating the potentially life-threating consequences of USUF vegan diets in children^54–62^.

### USUF vegan diets are sociologically implausible

Although ethical veganism is not inherently a Western concept, vegan diets appear largely to have been introduced to India^63,64^ and China^65^ from Western countries in the second half of the 20th century, with a notable increase in prevalence of vegan diets since the 2010s according to anecdoftal reports^66^. In India, vegan diets were still widely rejected by some vegetarian leaders in the 2000s. Although the prevalence of vegan diets in China and India may be increasing rapidly, it likely remains below 1%^67^ and possibly under 0.1% ^35^.

Despite occasional claims to the contrary^68–73^, there is no historical record of traditionally vegan communities in China, India, or elsewhere. While ASF-free diets were practiced by some individuals long before the term vegan was coined in 1944, these were likely rare exceptions. Vegan diets also still seem to have been rare in the second half of the 20th century in India despite its high vegetarian prevalence^74–87^. In India, traditional vegetarianism is largely motivated by religious doctrine rather than compassion or animal rights^6^. Although some religious leaders (e.g., Jains^88^) now promote veganism (contrary to tradition), national adoption likely remains low. Vegan diets break with Indian vegetarian tradition and, outside the modern vegan movement, are likely largely restricted to short-term ascetic practices. Similarly, Chinese Buddhist monastic diets may at times have been near-vegan, reflecting vegetarian diets with minimal intake of dairy and eggs^89^. However, a record of long-term vegan Buddhist populations in China is lacking. Although the existence of large numbers of vegans unconnected to the modern vegan movement is a possibility, the burden of proof should lie with those claiming their existence. Table 3 shows that the described studies did not assess vegan status in sufficient detail to confirm large numbers of vegans, even if involuntary.

While in some Western countries vegan diets are associated with higher education^43^, Table 6 shows that a substantial proportion of vegans in the described studies lacked even primary education, with many living under severe financial constraints. This suggests that many of these vegans were not voluntarily vegans (e.g., motivated by animal welfare or environmental/health views).

Additionally, the argument that many monetarily poor individuals cannot afford ASF is a generalization that may explain low but not absent ASF intake. Poor individuals without intentional vegan motives are unlikely to avoid all ASF and tend instead to maximize their involuntarily restricted ASF consumption. Evidence supports this: ASF intake rises whenever resources permit^90–94^, cash transfers increase ASF intake^95,96^, and rising incomes in China and India have drastically increased ASF consumption ^97,98^. Therefore, it may be assumed that poverty-constrained diets are rarely vegan over weeks or months, and if they are, they likely reflect globally deficient diets and global undernutrition^99^.

The present perspective does not argue that individuals forced by poverty to be involuntary vegans should not be classified as vegans (because of a lack of voluntariness or a righteous reason; Table 1). Rather, the proposed concept of poverty-induced involuntary vegan diets appears to be a theoretical abstraction rather than an observable phenomenon (outside of global undernutrition and starvation).

### Neither voluntary nor involuntary factors can explain the findings

If the high prevalences of vegan diets (Table 2) cannot plausibly be explained by voluntary choice or involuntary circumstances, the most likely explanation is that the reported prevalences are erroneous, potentially due to misclassification resulting from underassessment (insufficient tools) and/or underreporting. Notably, in individuals adhering to vegan or near-vegan USUF diets, underreporting may result from impaired recall ability due to vitamin B12 deficiency^100^.

### Differences between Western vegan cohorts and the cited studies from China and India

The lack of distinction between supplemented/fortified vegan diets and USUF vegan diets also applies to two long-running Western cohort studies: AHS-2 (USA and Canada) and EPIC-Oxford (UK). However, important differences exist in vegan status assessment between these studies and the cited studies from China and India. Table 4 and Table 5 summarize study characteristics indicating that dietary assessment in the cited studies from China and India was insufficient to infer the presence of vegan diets.

**Fig. 1:**
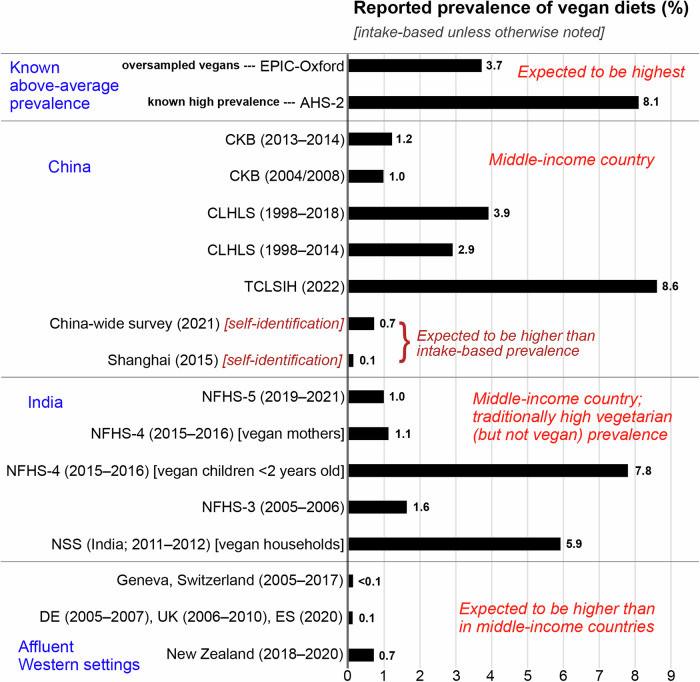
Prevalence of vegan diets (%) reported in the described studies. Prevalence refers to adults unless otherwise indicated. Further details can be found in the **Supplementary information**. AHS-2 Adventist Health Study 2, CKB China Kadoorie Biobank, CLHLS Chinese Longitudinal Healthy Longevity Survey, DE Germany, EPIC European Prospective Investigation into Cancer and Nutrition, ES Spain, NFHS National Family Health Survey; NSS National Sample Survey, TCLSIH Tianjin Chronic Low-grade Systemic Inflammation and Health, UK United Kingdom.

Figure 1 compares vegan diet prevalences reported in EPIC-Oxford, AHS-2, the cited studies, and several other studies. The highest prevalence (8.6%) was in TCLSIH, conducted in Tianjin near Beijing (2013–2019)^4^. This prevalence is unprecedented in the general population, comparable only to the 7–9% intake-defined vegans in AHS-2^101^. However, AHS-2 participants belong to a church that encourages vegan diets^102^, and long-term intake is assessed using a detailed FFQ with 27 items to assess vegan status (personal communication, G. Fraser, October 2025)^103^.

### Further confirmation of misclassification in the cited studies from China and India

Notably, discrepancies in vegan diet definitions is acknowledged in one of the cited publications on vegans in NFHS-3 (India), which states that vegans likely consumed dairy, particularly butter and ghee (Table 2)^8^. If so, this may also apply to NFHS-4 and NFHS-5. Discrepancies in vegan diet definitions are also present in the publication on vegan mothers and their children in NFHS-4, which states that maternal vegan status was reliably identified (as mothers were asked about usual diets) but also that the analysis was constrained by measurement limitations in identifying vegetarian diets and lack of data on individual food intakes^6^. Furthermore, the same publication reporting vegan diets in NFHS-4 states that “Clearly, 24 h recall cannot reliably be used to classify a child as vegetarian or not”^6^. The intake assessment window for young children in NFHS-4 was in fact less than 24 h: food items that the children may have consumed “in the day or night preceding the interview”^38^. Nevertheless, precisely this method was used to identify vegan children in the other cited paper on NFHS-4 ^7^ (Table 2), with these results subsequently cited in a position paper by the ESPGHAN Nutrition Committee on vegan children^27^. This ESPGHAN position paper also includes discrepant vegan diet definitions, referring to these children as vegan, while at the same time describing their diet as a “vegan-like [dietary] pattern”^27^. However, a 24-hour recall cannot identify either^6,104,105^. Additional limitations in dietary assessment among NFHS-4 children are summarized in Table 3. Furthermore, the prevalence of vegan children in NFHS-4 (7.8%; 2015–2016; Table 2)^7^ contrasts with data from the Comprehensive National Nutrition Survey (India, 2016–2018), which suggests that nearly 38% of children aged 2–4 years of lacto-vegetarian mothers did not consume dairy products in the previous 24 h^106^. Applying a methodology akin to the paper on NFHS-4 ^7^, this would suggest a vegan diet prevalence of about 17% (38% of about 44% lacto-ovo-vegetarian mothers^106^), which highlights the extent to which insufficient assessment can inflate vegan prevalence.

One cited paper reporting vegans in CLHLS (China) states that “the lack of data on dairy consumption precluded us from identifying the ovo-lacto-vegetarian group”^3^, while another states that non-assessment of dairy intake up to 2008 “could possibly lead to misclassification of different vegetarian dietary groups”^2^, confirming that CLHLS data are insufficient to identify vegan diets. Nevertheless, the same research group reported vegans in CLHLS in the two cited publications^2,3^ (Table 2), with one additional more recent publication^46^ and a further recent publication by another research group^47^. Furthermore, one of the articles acknowledges that CLHLS participants were asked about “food intake frequency at present instead of a given time range” which is “prone to information bias”^2^.

Authors reporting vegans in CKB (China) suggest that many of those classified as vegans likely adhered to a vegan diet involuntarily, i.e., they were forced to forgo ASF due to poverty^1^. They further highlight potential misclassification due to superficial dietary assessment and social desirability bias (underreporting). They note that 65% of baseline (2004) vegans were reclassified as omnivores at follow-up (2013)—only 12% as vegans—coinciding with improved socioeconomic status and additional assessment of cheese and milk powder in 2013^1^, suggesting underassessment at baseline. What the authors do not note is that the reported vegan prevalence (~1%; also observed in 2013; Table 2) and the extremely low educational levels among vegans appear implausible^1^ (Table 6).

### Incorrect usage of the term vegan and underassessment of ASF intake are common

Additional studies indicate that the term vegan is not always correctly understood by researchers. For example, one study (India) classified vegetarians who avoided eggs as “lacto-vegans”^107^, while another study (USA) classified those avoiding chicken and fish as “lacto-ovo-vegans”^108^.

Evidence also suggests that ASF intake in low-income settings is often underestimated^104,109,110^, partly because certain ASF are not assessed^92–94^. Consequently, the prevalence of ASF-free diets may easily be overestimated. For instance, studies in sub-Saharan Africa found prevalences of ASF-free diets exceeding 50% (based on intake data), yet authors did not describe these diets as vegan^92,111^. Moreover, some evidence suggests that ASF intake (possibly meat in particular) tends to be underreported^112^. Researchers should also take into account potential sociocultural factors which may strongly influence rates of misreporting and underassessment of ASF intake.

Underreporting is common, whereas vegan diets are uncommon. Therefore, even if underreporting is low at the group level^104^, if a small percentage (e.g., 3%) of participants strongly underreports intake, this may substantially inflate the estimated prevalence of ASF-free (inferred vegan) diets (e.g., from 0% to 2%).

## Conclusion

This perspective argues that many participants classified as vegans in the cited studies (Table 2) likely did not follow a vegan diet, as such high prevalences cannot be explained by voluntary choice or by involuntary, poverty-related complete ASF avoidance. Intake assessment was insufficient (Table 3), but even if adequate, implausible prevalences warrant scrutiny. Future studies may consider identifying vegans using a detailed FFQ (as in AHS-2) combined with self-identification (“my diet is vegan”). With the rising popularity of vegan diets, large cities in China and India may be highly suitable for recruiting large numbers of true vegans. Attention to variation in how vegan diets are understood by researchers and the public is recommended during study design, recruitment, data analysis, and reporting. Explicit, consistent definitions (whichever are chosen) are crucial to minimize misclassification and miscommunication of study findings.

## Supplementary information

Supplementary Information

## Data Availability

Not applicable.

## References

[1] Dunneram, Y. et al. Methods and participant characteristics in the Cancer Risk in Vegetarians Consortium: a cross-sectional analysis across 11 prospective studies. *BMC Public Health* **24**, 2095 (2024). 39095780 10.1186/s12889-024-19209-y PMC11296327

[2] Huang, Y. et al. Association between vegetarian diet and risk of frailty in Chinese older adults: a prospective study. *BMC Med.* **23**, 352 (2025). 40598166 10.1186/s12916-025-04232-6 PMC12211529

[3] Jigeer, G. et al. Vegetarian diet and healthy aging among Chinese older adults: a prospective study. *NPJ Aging* **11**, 25 (2025). 40169599 10.1038/s41514-025-00213-4 PMC11961757

[4] Bai, S. et al. Associations between dietary patterns and nephrolithiasis risk in a large Chinese cohort: is a balanced or plant-based diet better? *Food Funct.* **14**, 3220–3229 (2023). 36920109 10.1039/d2fo03993a

[5] Priya, S. & Thakur, R. Understanding gender variation in the risk factors of hypertension through cross sectional analysis. *Sci. Rep.* **15**, 33931 (2025). 41028039 10.1038/s41598-025-09865-4 PMC12484557

[6] Headey, D. D. & Palloni, G. Stunting and wasting among indian preschoolers have moderate but significant associations with the vegetarian status of their mothers. *J. Nutr.* **150**, 1579–1589 (2020). 32171005 10.1093/jn/nxaa042 PMC7269725

[7] Pandey, S. & Kashima, S. Effects of dairy intake on anthropometric failure in children ages 6 to 23 mo consuming vegetarian diets and fulfilling minimum dietary diversity in India. *Nutrition* **91–92**, 111446 (2021). 34587573 10.1016/j.nut.2021.111446

[8] Agrawal, S., Millett, C. J., Dhillon, P. K., Subramanian, S. V. & Ebrahim, S. Type of vegetarian diet, obesity and diabetes in adult Indian population. *Nutr. J.* **13**, 89 (2014). 25192735 10.1186/1475-2891-13-89 PMC4168165

[9] Mu, J., Agarwal, D. & Bharani, T. Environmental Sustainability and Chronic Disease Outcomes across Four Sustainable Dietary Patterns. *J. Nutr.* **156**, 101504 (2026). 41905733 10.1016/j.tjnut.2026.101504

[10] Merriam-Webster Dictionary. vegan. 2026. https://www.merriam-webster.com/dictionary/vegan (accessed 8 May 2026).

[11] Collins Dictionary. vegan. 2026. https://www.collinsdictionary.com/dictionary/english/vegan (accessed 8 May 2026).

[12] Dybvik, J. S., Svendsen, M. & Aune, D. Vegetarian and vegan diets and the risk of cardiovascular disease, ischemic heart disease and stroke: a systematic review and meta-analysis of prospective cohort studies. *Eur. J. Nutr.* **62**, 51–69 (2023). 36030329 10.1007/s00394-022-02942-8 PMC9899747

[13] Koeder, C. & Perez-Cueto, F. J. A. Vegan nutrition: a preliminary guide for health professionals. *Crit. Rev. Food Sci. Nutr.* **64**, 670–707 (2024). 35959711 10.1080/10408398.2022.2107997

[14] Vegan Society. Definition of veganism. 2026. https://www.vegansociety.com/go-vegan/definition-veganism (accessed 8 May 2026).

[15] Sanders T. A. B. Vegan/vegetarian diets. In: *Human Nutrition*. Oxford University Press, 2023. 10.1093/hesc/9780198866657.003.0021.

[16] Echiburu, N., Also-Fontanet, M. A., Sisó-Almirall, A. & González-de Paz, L. Impact of plant-based diets and associations with health, lifestyle and healthcare utilisation: a population-based survey study. *Public Health Nutr.* **28**, e120 (2025). 40670331 10.1017/S1368980025100669 PMC12465083

[17] Gimpfl S. et al. Self-reported adherence to vegetarian and vegan diets: insights from the 3rd bavarian food consumption survey. *Nutr. Bull*. 10.1111/nbu.70029. 10.1111/nbu.70029 PMC12621161 40926513

[18] Hedegaard, S., Nohr, E. A., Olsen, S. F., Halldorsson, T. I. & Renault, K. M. Adherence to different forms of plant-based diets and pregnancy outcomes in the Danish National Birth Cohort: a prospective observational study. *Acta Obstet. Gynecol. Scand.* **103**, 1046–1053 (2024). 38263894 10.1111/aogs.14778 PMC11103146

[19] Suzuki, K. et al. Repeated hypocalcemia in a patient with “Hikikomori” following veganism. *Heliyon* **8**, e09563 (2022). 35711977 10.1016/j.heliyon.2022.e09563 PMC9192804

[20] Aguirre, J. A. et al. Serious neurological compromise due to vitamin B12 deficiency in infants of vegan and vegetarian mothers. *Arch. Argent. Pediatr.* **117**, e420–e424 (2019). 31339288 10.5546/aap.2019.e420

[21] Gansweith, E. & Hoving, C. Exploring perceived social norms when considering a plant-based diet - an interview study. *Food Humanit.* **5**, 100858 (2025).

[22] Fuschlberger, M. & Putz, P. Vitamin B12 supplementation and health behavior of Austrian vegans: a cross-sectional online survey. *Sci. Rep.* **13**, 3983 (2023). 36949098 10.1038/s41598-023-30843-1 PMC10033911

[23] Salmivaara, L., Niva, M., Silfver, M. & Vainio, A. How vegans and vegetarians negotiate eating-related social norm conflicts in their social networks. *Appetite* **175**, 106081 (2022). 35569603 10.1016/j.appet.2022.106081

[24] Wozniak, H. et al. Vegetarian, pescatarian and flexitarian diets: sociodemographic determinants and association with cardiovascular risk factors in a Swiss urban population. *Br. J. Nutr.* **124**, 844–852 (2020). 32418548 10.1017/S0007114520001762 PMC7525113

[25] Tong, T. Y. N. et al. The plasma proteome of plant-based diets: Analyses of 2920 proteins in 49,615 people. *Clin. Nutr.* **53**, 144–154 (2025). 40912079 10.1016/j.clnu.2025.08.032

[26] Greenwell, J., Grant, M., Young, L., Mackay, S. & Bradbury, K. E. The prevalence of vegetarians, vegans and other dietary patterns that exclude some animal-source foods in a representative sample of New Zealand adults. *Public Health Nutr.* **27**, e5 (2023). 38050700 10.1017/S1368980023002677 PMC10830381

[27] Verduci, E. et al. Vegan diet and nutritional status in infants, children and adolescents: a position paper based on a systematic search by the ESPGHAN Nutrition Committee. *J. Pediatr. Gastroenterol. Nutr*. 10.1002/jpn3.70182. 10.1002/jpn3.70182 PMC12580465 40819279

[28] Harrison, L. et al. Planetary Health Diet in a hospital cafeteria: Increasing employee satisfaction and reducing greenhouse gas emissions and costs. *Z. Evid. Fortbild. Qual. Gesundhwes* **192**, 77–87 (2025). 39890584 10.1016/j.zefq.2024.12.003

[29] Hanslian, E. et al. Attitudes toward healthy nutrition in Germany - results from an online-representative cross-sectional survey. *Front Nutr.* **11**, 1480980 (2024). 39897537 10.3389/fnut.2024.1480980 PMC11783846

[30] Burke, D. T., Hynds, P. & Priyadarshini, A. Assessing the One Health (ecosystem, animal and human health) impacts of current dietary patterns based on farm-to-fork life cycle assessment in the Republic of Ireland. *Sci. Total Environ.* **975**, 179313 (2025). 40187334 10.1016/j.scitotenv.2025.179313

[31] Monori, G., Memon, A. & Archer, G. A cross-sectional study of the cost and nutritional content of plant-based meat-imitation products in supermarkets and plant-based products in restaurants in the United Kingdom. *Nutr. Health* **32**, 979–990 (2026). 40421482 10.1177/02601060251344449 PMC13144640

[32] Daas, M. C. et al. Diversity of dietary protein patterns across Europe - Impact on nutritional quality and environmental sustainability. *Curr. Res Food Sci.* **10**, 101019 (2025). 40151663 10.1016/j.crfs.2025.101019 PMC11946498

[33] Arango-Angarita, A., Unar-Munguía, M., Zepeda-Tello, R., Batis, C. & Rivera, J. A. Impact of healthy and sustainable diets on the mortality burden from cardiometabolic diseases and colorectal cancer in Mexican adults: a modeling study. *BMC Public Health* **25**, 2648 (2025). 40764988 10.1186/s12889-025-23988-3 PMC12326827

[34] Miassi, Y. E. et al. Socio-cultural and economic factors affecting the choice of food diet in West Africa: a two‑stage Heckman approach. *Discov. Food* **2**, 16 (2022).

[35] Mao, X. et al. Prevalence of vegetarians and vegetarian’s health dietary behavior survey in Shanghai. *Wei Sheng Yan Jiu* **44**, 237–241 (2015). 25997226

[36] CKB. Kadoorie Study of Chronic Disease in China (Questionnaire). Section 5.1. 2004. https://www.ckbiobank.org/study-resources/survey-data (accessed 5 Nov 2025).

[37] Government of India. Ministry of Health and Family Welfare. National Family Health Survey (NFHS-5), 2019–2021. INDIA REPORT. 2022. https://dhsprogram.com/pubs/pdf/FR375/FR375.pdf (accessed 27 Sept 2025).

[38] Government of India. Ministry of Health and Family Welfare. National Family Health Survey (NFHS-4), 2015–16. INDIA REPORT. 2017. https://dhsprogram.com/pubs/pdf/fr339/fr339.pdf (accessed 27 Sept 2025).

[39] Government of India. Ministry of Health and Family Welfare. National Family Health Survey (NFHS-3), 2005–2006. 2007. https://dhsprogram.com/pubs/pdf/frind3/frind3-vol1andvol2.pdf (accessed 17 Oct 2025).

[40] Cade, J., Thompson, R., Burley, V. & Warm, D. Development, validation and utilisation of food-frequency questionnaires - a review. *Public Health Nutr.* **5**, 567–587 (2002). 12186666 10.1079/PHN2001318

[41] Natrajan B., Jacob S. ‘provincialising’ vegetarianism putting Indian food habits in their place. 2018. https://publications.azimpremjiuniversity.edu.in/372/ (accessed 12 May 2026).

[42] Max Rubner-Institut, Bundesforschungsinstitut für Ernährung und Lebensmittel. Ergebnisbericht Teil 1. Nationale Verzehrsstudie II. 2008. https://www.mri.bund.de/fileadmin/MRI/Institute/EV/NVS_II_Abschlussbericht_Teil_1_mit_Ergaenzungsbericht.pdf (accessed 27 Sept 2025).

[43] forsa. Ernährungsreport 2023. Ergebnisse einer repräsentativen Bevölkerungsbefragung. 2023. https://www.bmleh.de/SharedDocs/Downloads/DE/_Ernaehrung/forsa-ernaehrungsreport-2023-tabellen.pdf (accessed 5 Nov 2025).

[44] Paslakis, G. et al. Prevalence and psychopathology of vegetarians and vegans - Results from a representative survey in Germany. *Sci. Rep.* **10**, 6840 (2020). 32321977 10.1038/s41598-020-63910-y PMC7176641

[45] GALLUP, Jones JM. In U.S., 4% Identify as Vegetarian, 1% as Vegan. 2023. https://news.gallup.com/poll/510038/identify-vegetarian-vegan.aspx (accessed 5 Nov 2025).

[46] Li, Y. et al. Vegetarian diet and likelihood of becoming centenarians in Chinese adults aged 80 y or older: a nested case-control study. *Am. J. Clin. Nutr.* **123**, 101136 (2026). 41391640 10.1016/j.ajcnut.2025.101136

[47] Song, Z. et al. A gradient risk of cognitive impairment with vegetarian diets in older adults: highest for vegan and potential benefit for pescatarian. *Food Res. Int.* **229**, 118455 (2026). 41763779 10.1016/j.foodres.2026.118455

[48] Koeder, C. Toward supplementation guidelines for vegan complementary feeding. *Food Sci. Nutr.* **12**, 10962–10971 (2024). 39723035 10.1002/fsn3.4565 PMC11666816

[49] Brocadello, F., Levedianos, G., Piccione, F., Manara, R. & Pesenti, F. F. Irreversible subacute sclerotic combined degeneration of the spinal cord in a vegan subject. *Nutrition* **23**, 622–624 (2007). 17616346 10.1016/j.nut.2007.05.006

[50] De Rosa, A. et al. Subacute combined degeneration of the spinal cord in a vegan. *Clin. Neurol. Neurosurg.* **114**, 1000–1002 (2012). 22316611 10.1016/j.clineuro.2012.01.008

[51] Huang, Q.-N. et al. Effect of roasted purple laver (nori) on vitamin B12 nutritional status of vegetarians: a dose-response trial. *Eur. J. Nutr.* **63**, 3269–3279 (2024). 39352476 10.1007/s00394-024-03505-9 PMC11519184

[52] Marques de Brito, B. et al. Vitamin B12 sources in non-animal foods: a systematic review. *Crit. Rev. Food Sci. Nutr.* **63**, 7853–7867 (2023). 35343314 10.1080/10408398.2022.2053057

[53] Koller, A. et al. Health aspects of vegan diets among children and adolescents: a systematic review and meta-analyses. *Crit. Rev. Food Sci. Nutr.* **64**, 13247–13258 (2024). 37811643 10.1080/10408398.2023.2263574

[54] Zmora, E., Gorodischer, R. & Bar-Ziv, J. Multiple nutritional deficiencies in infants from a strict vegetarian community. *Am. J. Dis. Child* **133**, 141–144 (1979). 105630 10.1001/archpedi.1979.02130020031005

[55] Shinwell, E. D. & Gorodischer, R. Totally vegetarian diets and infant nutrition. *Pediatrics* **70**, 582–586 (1982). 6812012

[56] David, J. & Fencl, F. Life-threatening Manifestations of Vitamin B12 Deficiency in Infants on a Vegan Diet. *Klin. Padiatr.* **233**, 306–307 (2021). 34470065 10.1055/a-1480-7938

[57] Ruggieri, V. L. & Arberas, C. L. Regresión autista: aspectos clínicos y etiológicos. *RevNeurol* **66**, 17 (2018). 29516448

[58] Barrantes, E. V., Medina, L. Z., Arredondo-Nontol, M. & Gomez, C. M. Hyperpigmentation in an Infant due to Vitamin B12 Deficiency: Case Report. *J. Paediatr. Child Health* **62**, 484–487 (2026). 41566957 10.1111/jpc.70294

[59] Singanamalla, B., Madaan, P., Saini, L. & Sankhyan, N. Vitamin B12 Deficiency: An Association or Etiology of Pseudotumor Cerebri in An Infant. *Indian J. Pediatr.* **87**, 658–659 (2020). 32036594 10.1007/s12098-020-03212-3

[60] Blasco-Alonso, J., Gil-Gómez, R., García Ruiz, A., Cortés Herrera, M. & Gutiérrez Schiaffino, G. Severe encephalopathy and vitamin B12 deficiency: reversibility after nutritional therapy. *Nutr Hosp* 2020. 10.20960/nh.03293. 10.20960/nh.03293 33241939

[61] Sharma, N. K. et al. Vitamin B12 deficiency in an infant with neurological and hematological findings: A case report. *Clin. Case Rep.* **11**, e7770 (2023). 37554579 10.1002/ccr3.7770 PMC10405240

[62] Kumar, P., Sukhija, J., Nagarajan, B. & Sankhyan, N. Infantile Vitamin B12 Deficiency with Reversible Acquired Vision Loss. *Indian J. Pediatr.* **91**, 310–310 (2024). 37658283 10.1007/s12098-023-04839-8

[63] Kotebagilu, N. P., Bhatia, S. & Piramanayagam, S. A qualitative investigation on Indian vegan food service providers’ perspective of trends, challenges and the future of vegan consumption. *Int. J. Gastronomy Food Sci.* **34**, 100824 (2023).

[64] Kumar, S. Veganism, Hinduism, and Jainism in India. A geo-cultural inquiry. In: *The Routledge Handbook of Vegan Studies*. Routledge: London, 2021. https://www.taylorfrancis.com/chapters/edit/10.4324/9781003020875-21/veganism-hinduism-jainism-india-saurav-kumar (accessed 27 Sept 2025).

[65] Cao, D. Chinese Takeaways: Vegetarian Culture in Contemporary China. 2018. 10.17863/CAM.42327.

[66] Narayan, S. *Satva. Essence of 53 vegan journeys*. Satvik Vegan Society: Byndoor, Karnataka, 2022.

[67] Chung, J. Y., Bryant, C. J. & Asher, K. E. Plant-based meats in China: a cross-sectional study of attitudes and behaviours. *J. Hum. Nutr. Diet.* **36**, 1090–1100 (2023). 36151900 10.1111/jhn.13092

[68] Wiwanitkit, V. Reticulocyte counts of Thai vegans compared with nonvegetarians. *Lab Hematol.* **13**, 39–40 (2007). 17353183

[69] Tarocco, F., Rossi, A., Zhang, B. W. & Francescon, S. Eating Like a Buddhist: Vegetarianism and Ethical Foodscapes in the Twenty-First Century. *AnnOr* 2024; **60**: JournalArticle_18156.

[70] Weeraperuma, S. *Jainism: Vegan Connect. Vegan* **Autumn 1990**, 9 (1990).

[71] Whittle, F. C. Impressions of an Indian journey. *Vegan* **Summer 1961**, 7 (1961).

[72] Sanderson, J. Editorial. *Vegan* **Summer 1970**, 41 (1970).

[73] Cobell, D. Letters to the editor. *Vegan* **Summer 1970**, 72 (1970).

[74] Dinshah H. J. *Song of India*. 1st edn American Vegan Society [pages 38, 72, 82]: Malaga, New Jersey, 1973.

[75] Welch H. *The Practice of Chinese Buddhism 1900-1950*. Harvard University Press [page 112]: Cambridge, 1967.

[76] Vegan Society News and announcements. *Vegan* **Autumn 1957**, 14 (1957).

[77] Vegan Society Correspondence Bureau. *Vegan* **Winter 1958**, 35 (1958).

[78] Vegan Society Announcements. *Vegan* **Spring 1958**, 22 (1958).

[79] Vegan Society. Back numbers of ‘The Vegan’. *The Vegan* 1961; **Winter 1961**: 25.

[80] Clarke, V. & Maneka, G. Vegan Society patron. *Vegan* **Winter 2002**, 7 (2002).

[81] Vegan SocietyNews of members. A.S.R Chari, India. Vegan Autumn 1973, 20 (1973).

[82] BJ, G. unn-K. ing A vegan at the 19th World Vegetarian Congress. *Vegan* **Spring 1968**, 9 (1968).

[83] Chari, A. S. R. Benefits from veganism and nature cure. *Vegan* **Summer 1956**, 3–4 (1956).

[84] S. F. Book Review. *The Vegan* 1956; **Summer 1956**: 13.

[85] Desai, D. C. The religion of compassion. *Vegan* **Summer 1956**, 7–8 (1956).

[86] Vegan Society. The Vegan Society of India. *The Vegan* 1958; **Summer 1958**: 18.

[87] Vegan Society. The vegan dinner. *The Vegan* 1953; **Summer 1953**: 22.

[88] Seetharaman, H. Beyond Ethics: A Study of Attitudes Towards Animal Cruelty Among Jain Vegans and Non-Jain Vegans. *Confluence: J. Interdiscipl. Stud.* 2024; **VII–VIII**. https://cjids.in/beyond-ethics-a-study-of-attitudes-towards-animal-cruelty-among-jain-vegans-and-non-jain-vegans/ (accessed 8 Nov 2025).

[89] Welch, H. *The Practice of Chinese Buddhism 1900-1950*. Harvard University Press [page 113]: Cambridge, 1967.

[90] Headey, D. D., Ecker, O., Comstock, A. R. & Ruel, M. T. Poverty, price and preference barriers to improving diets in sub-Saharan Africa. *Glob. Food Sec.* **36**, 100664 (2023). 36937376 10.1016/j.gfs.2022.100664 PMC10015269

[91] Humphries, D. L. et al. Households across all income quintiles, especially the poorest, increased animal source food expenditures substantially during recent Peruvian economic growth. *PLoS One* **9**, e110961 (2014). 25372596 10.1371/journal.pone.0110961 PMC4220962

[92] Mekonen, E. G. Animal source food consumption and its determinants among children aged 6 to 23 months in sub-Saharan African countries: a multilevel analysis of demographic and health survey. *BMC Public Health* **24**, 2060 (2024). 39085814 10.1186/s12889-024-19628-x PMC11290212

[93] Miller, V. et al. Global, regional, and national consumption of animal-source foods between 1990 and 2018: findings from the Global Dietary Database. *Lancet Planet Health* **6**, e243–e256 (2022). 35278390 10.1016/S2542-5196(21)00352-1 PMC8926870

[94] Darapheak, C., Takano, T., Kizuki, M., Nakamura, K. & Seino, K. Consumption of animal source foods and dietary diversity reduce stunting in children in Cambodia. *Int Arch. Med* **6**, 29 (2013). 23866682 10.1186/1755-7682-6-29 PMC3720190

[95] Manley, J., Alderman, H. & Gentilini, U. More evidence on cash transfers and child nutritional outcomes: a systematic review and meta-analysis. *BMJ Glob. Health* **7**, e008233 (2022). 35365481 10.1136/bmjgh-2021-008233 PMC8977747

[96] Tonguet-Papucci, A. et al. Unconditional seasonal cash transfer increases intake of high-nutritional-value foods in young Burkinabe children: results of 24-hour dietary recall surveys within the Moderate Acute Malnutrition Out (MAM’Out) randomized controlled trial. *J. Nutr.* **147**, 1418–1425 (2017). 28566529 10.3945/jn.116.244517

[97] Popkin, B. M. Synthesis and implications: China’s nutrition transition in the context of changes across other low- and middle-income countries. *Obes. Rev.* **15**, 60–67 (2014). 24341759 10.1111/obr.12120 PMC3869101

[98] Rampal, P. An analysis of protein consumption in India through plant and animal sources. *Food Nutr. Bull.* **39**, 564–580 (2018). 30482046 10.1177/0379572118810104

[99] Ellis, F. R., Powell, M. E. & Kurtha, A. N. Death after vegan diet. *Lancet* **2**, 44–45 (1968). 4173254 10.1016/s0140-6736(68)92909-7

[100] Blom, J. D. Hallucinations and vitamin B12 deficiency: a systematic review. *Psychopathology* **57**, 492–503 (2024). 39047712 10.1159/000540003 PMC11651228

[101] Teixeira Martins, M. C., Jaceldo-Siegl, K., Fan, J., Singh, P. & Fraser, G. E. Short- and long-term reliability of adult recall of vegetarian dietary patterns in the Adventist Health Study-2 (AHS-2). *J. Nutr. Sci.* **4**, e11 (2015). 26097699 10.1017/jns.2014.67 PMC4462762

[102] McBride, D. C. et al. Health beliefs, behavior, spiritual growth, and salvation in a global population of seventh-day adventists. *Rev. Religious Res.* **63**, 535–557 (2021).

[103] Jaceldo-Siegl, K. et al. Validation of nutrient intake using an FFQ and repeated 24 h recalls in black and white subjects of the Adventist Health Study-2 (AHS-2). *Public Health Nutr.* **13**, 812–819 (2010). 19968897 10.1017/S1368980009992072 PMC3417357

[104] Gibson, R. S., Charrondiere, U. R. & Bell, W. Measurement errors in dietary assessment using self-reported 24-hour recalls in low-income countries and strategies for their prevention. *Adv. Nutr.* **8**, 980–991 (2017). 29141979 10.3945/an.117.016980 PMC5683000

[105] Knüppel, S., Norman, K. & Boeing, H. Is a single 24-hour dietary recall per person sufficient to estimate the population distribution of usual dietary intake? *J. Nutr.* **149**, 1491–1492 (2019). 31172195 10.1093/jn/nxz118

[106] MoHFW. Comprehensive National Nutrition Survey (CNNS) 2016-2018. National Report [Table 4.4; Figure 3.5]. 2019. https://www.unicef.org/india/media/2646/file/CNNS-report.pdf (accessed 5 Nov 2025).

[107] Joshi, O. V., Savale, R. R., Nalage, D., Biradar, A. & Sontakke, T. Lifestyle in flux: urbanization, dietary shifts, and endocrine health in emerging adulthood. *Reprod. Biol. Endocrinol.* **23**, 118 (2025). 40847396 10.1186/s12958-025-01442-8 PMC12372347

[108] Perry, C. L., McGuire, M. T., Neumark-Sztainer, D. & Story, M. Adolescent vegetarians: how well do their dietary patterns meet the healthy people 2010 objectives?. *Arch. Pediatr. Adolesc. Med* **156**, 431–437 (2002). 11980547 10.1001/archpedi.156.5.431

[109] Casey, E. M. D., Mojarrabi, M., Hannan-Jones, M. T. & Bogard, J. R. Measuring dietary intake in low-and middle-income countries: a systematic review of the methods and tools for estimating fish and seafood intake. *Nutr. Rev.* **82**, 453–466 (2024). 37335872 10.1093/nutrit/nuad067 PMC10925904

[110] Berti, P. R., Fallu, C. & Cruz Agudo, Y. A systematic review of the nutritional adequacy of the diet in the Central Andes. *Rev. Panam. Salud Publica* **36**, 314–323 (2014). 25604101

[111] Kedir, S. et al. Animal source food consumption and anaemia among school adolescent girls in Silti District, Central Ethiopia: a public health perspective. *J. Nutr. Sci.* **13**, e89 (2024). 39703892 10.1017/jns.2024.76 PMC11658937

[112] Kurpad, A. V. & Thomas, T. Protein quality and its food source in the diets of young Indian Children. *J. Nutr.* **150**, 1350–1351 (2020). 32359143 10.1093/jn/nxaa100

[113] C. K. B. Kadoorie Study of Chronic Disease in China (Resurvey Questionnaire). 2008. https://www.ckbiobank.org/study-resources/survey-data (accessed 5 Nov 2025).

[114] CKB. China Kadoorie Biobank (2nd Resurvey Questionnaire). Version 2.2, CKB/ICC/2013. 2013. https://www.ckbiobank.org/study-resources/survey-data (accessed 5 Nov 2025).

[115] Borude, S. Which Is a Good Diet-Veg or Non-veg? Faith-based vegetarianism for protection from obesity-a myth or actuality?. *Obes. Surg.* **29**, 1276–1280 (2019). 30604082 10.1007/s11695-018-03658-7

[116] Qin, C. et al. The Relative Validity and Reproducibility of Food Frequency Questionnaires in the China Kadoorie Biobank Study. *Nutrients* **14**, 794 (2022). 35215443 10.3390/nu14040794 PMC8879142

[117] Davey, G. K. et al. EPIC-Oxford: lifestyle characteristics and nutrient intakes in a cohort of 33 883 meat-eaters and 31546 non meat-eaters in the UK. *Public Health Nutr.* **6**, 259–269 (2003). 12740075 10.1079/PHN2002430

[118] Oxford Population Health (CEU). Questionnaires. 2025. https://www.ceu.ox.ac.uk/research/epic-oxford-1/about-the-study/questionnaires (accessed 5 Nov 2025).

