# Supplementary material for: Inferred vegans in studies not designed to assess vegan status may be largely artifacts
Source: NPJ Sci Food. 2026 Jul 21;10:227. doi: 10.1038/s41538-026-00985-3 (PMC13389288; doi:10.1038/s41538-026-00985-3)
Supplement: Supplementary file 1 — Supplementary Information [file 41538_2026_985_MOESM1_ESM.docx]

***Supplementary material***

Inferred vegans in studies not designed to assess vegan status may be largely artifacts

**Prevalence of vegan diets in China**

Two more recent papers on CLHLS suggest a vegan diet prevalence that is even higher than 2.9% ^1^ and 3.9% ^2^ (**Table 2**), namely 8.1% ^3^ (general CLHLS population) and 5.1% (CLHLS participants adults aged ≥80 years).^4^ The China-wide online survey (2021) included in **Figure 1** was a cross-sectional online survey (n = 1,206) with a vegan diet prevalence of 0.7% (based on self-identification); participants were adults, of whom 83% had post-secondary education; “vegan” was described as “only plant-based foods”. The latter two factors may overestimate vegan diet prevalence as high education may be associated with a higher vegan prevalence and because not all plant-*based* foods are vegan.^5^ The the Shanghai survey (published 2015) included in **Figure 1** reported 0.1% self-identified vegans ^6^; vegans were older, had lower formal education, and were more likely to be Buddhists.^6^ Nevertheless, wealth, education levels, and prevalence of Buddhism in this Shanghai sample were likely higher than in the CKB sample. Prevalence of vegan diets may be higher among Buddhists (and possibly Daoists) in China, though precise data appear to be lacking. Most lay Buddhists and lay Daoists in China do not appear to be vegetarians, much less vegans.^7,8^ Some authors suggest 4% of Buddhists in China are dietary vegans (which may be an overestimate) ^8^; assuming this vegan prevalence and a prevalence of Buddhism in China of <5% ^9^, the theoretical prevalence of Buddhist dietary vegans in China would be <0.2%. Data on the proportion of Buddhists among vegans in China are lacking.

1 Jigeer G, Wang K, Lv Y, Tucker KL, Shen X, Chen F *et al.* Vegetarian diet and healthy aging among Chinese older adults: a prospective study. *NPJ Aging* 2025; **11**: 25.

2 Huang Y, Jigeer G, Lv Y, Wang K, Ma X, Zou J *et al.* Association between vegetarian diet and risk of frailty in Chinese older adults: a prospective study. *BMC Med* 2025; **23**: 352.

3 Song Z, He Z, Wang X, Lei C, Ding M, Sun Z *et al.* A gradient risk of cognitive impairment with vegetarian diets in older adults: highest for vegan and potential benefit for pescatarian. *Food Res Int* 2026; **229**: 118455.

4 Li Y, Wang K, Lv Y, Jigeer G, Huang Y, Shen X *et al.* Vegetarian diet and likelihood of becoming centenarians in Chinese adults aged 80 y or older: a nested case-control study. *Am J Clin Nutr* 2026; **123**: 101136.

5 Chung JY, Bryant CJ, Asher KE. Plant-based meats in China: a cross-sectional study of attitudes and behaviours. *J Hum Nutr Diet* 2023; **36**: 1090–1100.

6 Mao X, Shen X, Tang W, Zhao Y, Wu F, Zhu Z *et al.* [Prevalence of vegetarians and vegetarian’s health dietary behavior survey in Shanghai]. *Wei Sheng Yan Jiu* 2015; **44**: 237–241.

7 Cao D. Chinese Takeaways: Vegetarian Culture in Contemporary China. 2018. doi:10.17863/CAM.42327.

8 Tseng AA. Equivalent Reduction in Greenhouse Gas Emissions by Mahayana Buddhists Practicing Vegetarian Diets. *J Relig Health* 2020; **59**: 598–613.

9 Pew Research Center. Measuring Religion in China: 3. Buddhism. 2023.https://www.pewresearch.org/religion/2023/08/30/buddhism/ (last accessed 16 July 2026).
